# Parental alienation diagnosis. A modern and effective subtype of domestic violence, endemic in Italian courts

**DOI:** 10.1186/1824-7288-40-S1-A34

**Published:** 2014-08-11

**Authors:** Maria Serenella Pignotti

**Affiliations:** 1 Dipartimento Feto-neonatale, AOU Meyer-University of Firenze, Viale Pieraccini 50100 Firenze Italy

## 

In 1985 Gardner, publishing on sexual abuse [1], described a clinical picture, Parental Alienation Syndrome (PAS) as a “*disorder that arises primarily in the context of child-custody disputes. Its primary manifestation is the child’s campaign of denigration against a good, loving parent, a campaign that has no justification.”*[2]. Originally Gardner defined PAS in gender specific terms, mothers as alienators and fathers as victims. Later, he claimed that either parent could be an alienator [2]. However, almost always, PAS affects women. Gardner specified “*when true parental abuse and/or neglect is present the child’s animosity may be justified, and so the parental alienation syndrome diagnosis is not applicable*” [2] but never clarified how to make such differential diagnosis [3-5]. In Gardner’s mind, women involved in divorce trial become psychopathic in the sphere of life related to parenting [6], although PAS “*is not only simply a matter of brainwashing or programming in that the children contribute their own elements*” [4]. It is based on three main principles: a. the children are liars; b. the father’s alienation is a mother’s responsibility; c. the allegations of maltreatment are false. To a large extent, PAS tautologically presumes lack of justification for the refusal, maternal programming and absence of children’s credibility. PAS’ treatment, based on legal coercion through court-ordered threats of deprivation of custody to force mothers and children to act with affection toward the father, violates the principles of good medical practices [7]. PAS’s theoretical roots lie on Gardner’s theory of human sexuality that justifies adult-child sexual contact and gender violence as beneficial to the reproduction of the species and PAS becomes “*a defence strategy for abusive fathers, facilitating these men’s projection of blame for their children’s refusal onto mothers as a counter-claim to, and evidentiary shield against, allegations of violence*” in domestic violence cases [7]. PAS is detrimental to children, women and honest men’ civil rights and to Justice! It is a tool aimed to punish women and children reacting to a patriarchical system that presumes all reports of male violence are false and punishes protective mothers. According to American Academy of Paediatrics family violence is a paediatric issue[8]. PAS-ideology’s influence on family and criminal Court could lead to wrong decisions with foreseeable emotional upset and trauma to the children and consequently to stark outcomes, including murder and suicide, as in the past [6].
